# Identification of microbiota in peri-implantitis pockets by matrix-assisted laser desorption/ionization time-of-flight mass spectrometry

**DOI:** 10.1038/s41598-018-37450-5

**Published:** 2019-01-28

**Authors:** Hwey-Chin Yeh, Jang-Jih Lu, Shih-Cheng Chang, Mao-Cheng Ge

**Affiliations:** 1 Linyeh Dental Clinic, Taipei, Taiwan; 2 Department of Periodontics, Section of Dentistry, Chang Gung Memorial Hospital Linkou Medical Center, Taoyuan, Taiwan; 3 Chang Gung University College of Medicine, Taoyuan, Taiwan; 4 Department of Laboratory Medicine, Chang Gung Memorial Hospital Linkou Medical Center, Taoyuan, Taiwan; 5 Department of Medical Biotechnology and Laboratory Science, Chang Gung University, Taoyuan, Taiwan

## Abstract

The purpose of this study was to identify the microbial communities that colonize peri-implantitis pockets using matrix-assisted laser desorption/ionization time-of-flight mass spectrometry (MALDI-TOF MS). Subjects having at least one implant with peri-implantitis, no diabetes, and not taking antibiotics in the previous 3 months were selected. Peri-implantitis was defined when surrounding bone loss ≥0.5 mm and bleeding on probing was found. Microbial samples were collected from peri-implantitis pockets using paper points. After incubation and isolation, the colonies were analyzed by MALDI-TOF MS. A total of 126 isolates were cultivated and identified from 12 samples, in identification rates of 82.5% at the species level and 12.72% at the genus level. Although the compositions were highly variable, major habitants in different peri-implant pockets could be identified. Among them the most distinguished were *Neisseria flavescens* (87%), *Streptococcus constellatus* (56%), *Slackia exigua* (46%), *Streptococcus intermedius* (45%), *Fusobacterium nucleatum* (45%) and *Gemella morbillorum* (43%). This preliminary study provides comprehensive and reliable data for future study designs involving MALDI-TOF MS and peri-implantitis in a more specific, easy, rapid and economical way. MALDI-TOF MS could be a new clinical method to evaluate and monitor oral microbiota associated with the disease.

## Introduction

Peri-implantitis is defined as an irreversible inflammatory reaction associated with loss of supporting bone around an osseointegrated implant in function. The prevalence of peri-implantitis has been inconsistently reported and was found in 26% to 56% of subjects and in 12% to 43% of implant sites^1–3^. Diagnosis of peri-implantitis is made in a similar way to that of periodontitis. It is detected both clinically and radiographically, including bleeding on probing and loss of supporting bone. The correct diagnosis and treatment of peri-implant disease is critical to avoid complete loss of osseointegration and implant loss^4,5^.

Essential evidence supports the view that microorganisms play a major role in causing peri-implantitis^6^. We currently understand that the microbiota associated with peri-implantitis is more complex than that found under healthy peri-implant conditions^7^. Patients with peri-implantitis harbor high levels of periodontal pathogens^8^. However, the microbiota of peri-implantitis is more diverse than that of periodontitis^9,10^.

Microbiologic methods used to study the presence of microorganisms in peri-implantitis sites seem to influence results of microbial profile studies. Microbiological results by both culture and checkerboard analysis only detect specific target bacteria. They are not practical for identifying the true diversity of potential pathogens and fail to correspond fully to the severity of the disease in terms of magnitude^11,12^. Currently, microorganisms are best identified using polymerase chain reaction (PCR) amplification of conserved regions of the 16S ribosomal RNA (rRNA) gene followed by clone library construction, which allows for the detection of previously uncultivated and unknown bacteria^9^. However, it is often expensive and time consuming, and quantitative results might be misleading because live and dead bacteria cannot be distinguished.

Matrix-assisted laser desorption/ionization time-of-flight mass spectrometry (MALDI-TOF MS) has been used extensively as a research tool for protein analysis and has emerged as a potential tool for microbial identification and diagnosis^13,14^. It is a soft ionization method, which allows for the desorption of peptides and proteins from both whole different cultured bacteria and crude bacterial extracts^15^. Ions are separated and detected according to their molecular masses and charges. Each mass peak corresponds to a molecular fragment released from a microbiological sample during laser desorption^16^. Bacteria can then be identified by comparing their mass spectrum with those obtained from known reference strains using multivariate statistical analysis^17^. Compared with other identification methods, MALDI-TOF MS shows a rapid turn-around time, low sample volume requirements and modest reagent costs^18^.

The purpose of the present study was to identify the microbiota of peri-implantitis pockets using a different but well-established method, MALDI-TOF MS, and to assess the composition of the cultivated microflora in peri-implantitis pockets.

## Methods

### Subjects and examinations

Patients with peri-implantitis were recruited in the periodontal department of Chang Gung Memorial Hospital Linkou Medical Center from June, 2014 to March, 2015. The enrollment criteria of implant subjects were as follows: (1) patient must have had at least one dental implant, (2) the implant of the patient must have had definitive prostheses for at least one year, and (3) the implant of the patient was diagnosed with peri-implantitis, as evident in bleeding on probing (BOP) and loss of supporting bone^2^. The exclusion criteria were: (1) patients who had taken any antibiotics within 3 months prior to clinical examination and sampling, (2) patients who had poorly controlled diabetes mellitus (HbA1c ≥8.0)^19,20^.

Information regarding patient profiles, such as age and gender, smoking habit, and medical history, such as disease and medication, were collected. The assessment of peri-implant health was based on clinical and radiographic examinations. The following variables were recorded at the mesial, buccal, distal, and lingual aspects of each implant: (1) pocket probing depth (PPD) measured in mm using a manual periodontal probe (Hu-Friedy PCPUNC 15 Mfg Co. Inc. Chicago IL), (2) BOP within 15 seconds following pocket probing, (3) suppuration within 15 seconds following pocket probing.

Intraoral radiographs were obtained using the long-cone parallel technique. The inter-thread pitch distance reported by the manufacturer or the length of the implant was used for the calibration of the “apical-coronal” measurements in each radiograph. Landmarks were chosen, and the position of the marginal bone was assessed. The distance to the crestal bone was measured, calculated and rounded to the nearest 0.5 mm at the mesial and distal aspects of the implant. Bone loss was detected by comparing the measurements of radiographs obtained at the examination and at the baseline when the prosthesis was connected. In cases with no available baseline radiographs, marginal bone levels located >2 mm apical of a reference landmark were registered as bone loss.

Peri-implantitis was defined when there was detectable bone loss (>0.5 mm, exceeding the measurement error) and BOP^2^. In subjects in which more than one implant met the inclusion criteria, only the implant with the most severe condition was studied, but all implants of the patients with peri-implant disease were treated.

### Ethics

Written informed consents were obtained from all subjects following the guideline of the Declaration of Helsinki. This study was independently reviewed and approved by the Ethics Committee of the Institutional Review Board of Chang Gung Medical Foundation (No. 102-3687A3).

### Sample collection and bacterial isolation and quantification

Microbial samples were collected from the peri-implantitis pockets at the following visit. Prior to sampling, the supra-gingival plaque was removed with a sterile curette. The implant abutments or prostheses were isolated with cotton rolls and gently dried with an air syringe. Subgingival samples were obtained by inserting #40 sterilized paper points into the peri-implant pockets at the mesial, buccal, distal and lingual aspects of each implant, until resistance was felt and seated for 30 seconds. The paper points were then removed and immediately placed in a sterile Eppendorf tube prepared with transportation medium of 1 ml sterile distilled water.

The samples were transported to the laboratory at 23 °C room temperature and processed within 30 minutes after sampling. Each sample was vortexed and then diluted 10-fold five times with 90% sodium chloride. Each 50 µl of the diluted sample solution of original samples, 10^2^-fold dilution and 10^5^-fold dilution was pipetted into four different agar plates, including anaerobe 5% sheep blood agar (CDC, BD BBL prepared plated media; Becton, Dickinson and Co., Sparks, MD), phenylethyl alcohol blood agar (BD BBL prepared plated media; Becton, Dickinson and Co., Sparks, MD) for anaerobic culture and Columbia CNA (colistin alidixic acid) agar (BD BBL prepared plated media; Becton, Dickinson and Co., Sparks, MD) and Trypticase soy blood agar (BD BBL prepared plated media; Becton, Dickinson and Co., Sparks, MD) for aerobic culture. These agar plates were incubated for 3 days to identify and quantify the colony-forming units (CFU) per milliliter according to the CFU per plate and dilution factor.

### Bacterial identification by MALDI-TOF MS

All organisms in all different morphotypes were identified by MALDI-TOF MS. A single colony was inoculated on a steel MALDI target plate by picking with a toothpick directly to a thin film. The microbial film was then overlaid with 1 μl 70% formic acid. After drying, the microbial film was then overlaid with 1 μl matrix solution (50% acetonitrile contain 1% α-cyano-4-hydroxycinnamic acid and 2.5% trifluoroacetic acid) and allowed to dry at room temperature. Mass spectra were acquired using the MALDI-TOF MS spectrometer (Microflex; Bruker Daltonics Inc., Billerica, MA, USA) in linear positive mode. MALDI-TOF MS analysis was performed in the automatic mode, and the maximum 240 laser shots were collected for each isolate. Bacterial test standard (BTS) (part no. 255343, Bruker Daltonics Inc.) was used in each run as a calibrator and for quality control. Measured mass spectra ranged from 2,000 to 20,000 Da. Extraction of the peaks from the generated mass spectra and their matching against the reference spectra of the integrated database containing 4613 MSP provided by the manufacturer was performed with MALDI Biotyper 3.1 software (Bruker Daltonics Inc.). The score value was defined by three components, the matches of the unknown spectrum against the main spectrum, the matches of the main spectrum peaks against the unknown spectrum, and the correlation of intensities of the matched peaks. When the score was between 2.0 and 1.7, the identification was considered enough confidence to the success of the genus level. The final score of <1.7 was considered failure or an ambiguous identification^18^.

### Statistical analysis

Microbial diversity from each implant was summarized and displayed as descriptive data only. The percentages of microorganisms were calculated based on the individual implant. The occurrence of each strain was counted. The microbial load and the counts of each strain were presented as medians, 25% and 75% quartiles. The median counts of each strain among different PPDs were calculated. The median counts and median percentages of different microbial types related to the deepest PPDs were also calculated. Nonparametric Kruskal-Wallis and Wilcoxon-Mann-Whitney tests were used to compare the differences in the median and spread of the microbial counts and percentages among different PPDs. All tests appear as two-sided *p* values, and they were declared statistically significant at *p* < 0.05.

## Results

### Population of the study

Twelve samples were collected from 12 peri-implantitis pockets in 12 patients. They included 6 females and 6 males, aged from 46 to 73 years with a mean of 60.0 ± 7.9 years. All patients had a history of moderate or advanced periodontitis. Two were current smokers, 2 were former smokers and the rest were non-smokers. Four were systemically healthy, but 8 were mildly compromised and medicated. All implants had rough surfaces and were located at the posterior sextants except one. Six implants were placed in the maxilla and the other six were in the mandible.

### Identification of microbial strains

**Figure 1:**
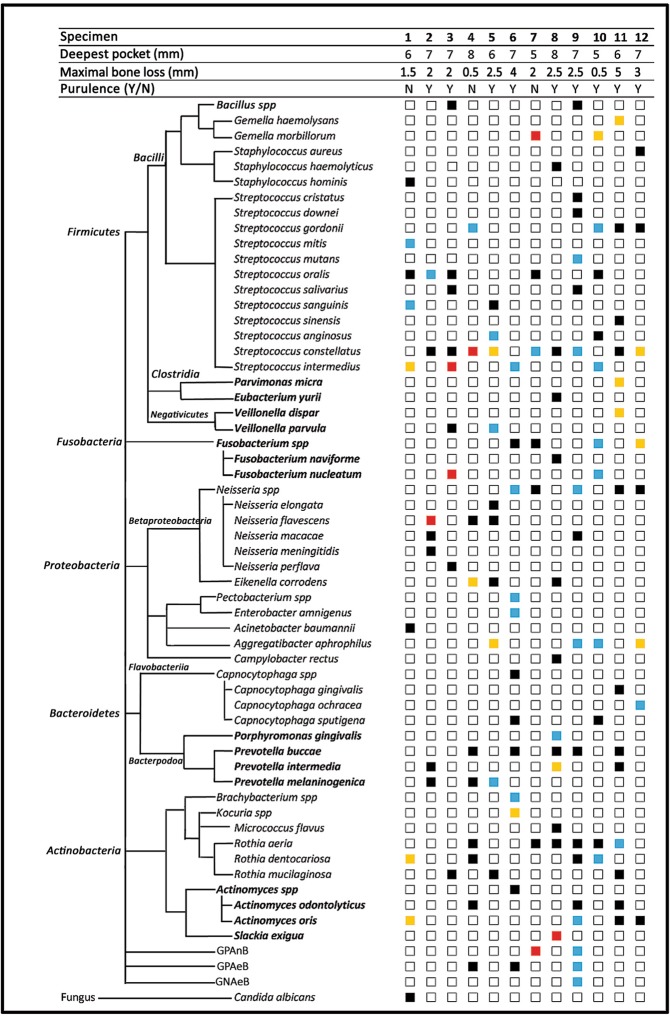
Microbial strains detected by MALDI-TOF MS from peri-implantitis pockets are shown in phylotypes. The percentages of microorganisms were calculated based on the individual sample. The percentages (p) of microbiota in each specimen were arbitrarily divided into different ranges and were marked with different colors, including black (p ≤ 5%), blue (5% < p ≤ 20%), yellow (20% < p ≤ 40%) and red (p > 40%).

A total of 126 isolates were cultivated from the 12 samples and sent for MALDI-TOF MS identification. Among them, 104 (82.5%) isolates were identified in species, 16 isolates (12.7%) were in genera and 6 (4.8%) were undefined. This resulted in identification rates of 82.5% at the species level and 95.2% at the genus level. Forty-seven species and 8 genera of bacteria were identified. These bacteria belonged to 5 phyla, including *Firmicutes*, *Fusobacteria*, *Proteobacteria*, *Bacteroidetes* and *Actinobacteria*. The 6 undefined isolates were all bacilli, comprising 2 Gram (+) anaerobic (GPAnB), 3 Gram (+) aerobic (GPAeB) and 1 Gram (−) aerobic (GNAeB) bacillus. Other than these, one isolate was a fungus, *Candida albicans* (Fig. 1).

### Microbial compositions

The numbers of microbial strains detected from each sample ranged from 7 to 17, with a median of 10. The compositions of individual samples are presented in Fig. 1. The percentages of microorganisms were calculated based on the individual sample. The percentages of microbiota in each sample were arbitrarily divided into different ranges and were marked with four colors, including black (≤5%), blue (5–20%), yellow (20–40%) and red (≥40%). Although the compositions were highly variable, some species (shown in red squares) could be identified as major habitants in different peri-implant pockets, such as *Neisseria flavescens* (87%), *Streptococcus constellatus* (56%), *Slackia exigua* (46%), *Streptococcus intermedius* (45%), *Fusobacterium nucleatum* (45%), *Gemella morbillorum* (43%) and one unknown gram-positive anaerobic *Bacillus* (43%).

### Microbial loads and occurrences

The total microbial counts ranged from 4.6 × 10^4^ to 3.1 × 10^6^ CFU/ml, with a median of 3.0 × 10^5^ CFU/ml (25%: 2.0 × 10^5^ CFU/ml, 75%: 6.6 × 10^5^ CFU/ml). The occurrences, median counts, 25% and 75% quartiles of microbial strains were listed in Table 1. *Streptococcus constellatus* was the most frequently detected species, found in 9 out of 12 samples. It was followed by *Rothia aeria*, *Streptococcus oralis* and *Prevotella buccae*. *Neisseria spp*. were detected in 5 samples but might represent different species. Twenty-eight microorganisms were repeatedly detected in different samples, whereas 31 out of 59 microorganisms (52.5%) were only found once.

**Table 1:** Microbial strains detected from peri-implant pockets by MALDI-TOF MS, in a decreasing order of occurrences out of 12 samples.

*Microbial strains*	Occurences (out of 12)	Counts (×10^3^ CFU/ml)			*Microbial strains*	Occurences (out of 12)	Counts (×10^3^ CFU/ml)
		Median	25%	75%			Median
*Streptococcus constellatus*	9	5.0	1.0	90.0	***Fusobacterium naviforme***	1	30.0
*Rothia aeria*	6	2.0	0.3	8.3	***Parvimonas micra***	1	100.0
***Prevotella buccae***	5	1.0	0.3	2.0	***Porphyromonas gingivalis***	1	300.0
*Streptococcus oralis*	5	6.0	3.4	6.0	***Slackia exigua***	1	1000.0
*Neisseria spp*	5	10.0	4.0	22.0	***Veillonella dispar***	1	100.0
***Actinomyces oris***	4	12.5	8.0	18.8	*Gemella haemolysans*	1	100.0
***Fusobacterium spp***	4	40.0	22.5	50.0	*Staphylococcus aureus*	1	7.0
*Streptococcus gordonii*	4	15.0	8.8	22.5	*Staphylococcus haemolyticus*	1	0.1
*Streptococcus intermedius*	4	100.0	82.5	200.0	*Staphylococcus hominis*	1	5.0
*Aggregatibacter aphrophilus*	4	40.0	27.5	112.5	*Streptococcus cristatus*	1	10.0
*Rothia dentocariosa*	4	21.0	1.8	42.5	*Streptococcus downei*	1	10.0
***Actinomyces odontolyticus***	3	1.0	1.0	1.5	*Streptococcus mitis*	1	9.0
***Prevotella intermedia***	3	1.0	0.9	350.5	*Streptococcus mutans*	1	30.0
***Prevotella melaninogenica***	3	5.0	3.0	52.5	*Streptococcus sinensis*	1	1.0
*Eikenella corrodens*	3	50.0	30.0	75.0	*Acinetobacter baumannii*	1	0.2
*GPAeB* ^a^	3	20.0	15.0	60.0	*Brachybacterium spp*	1	200.0
*Neisseria flavescens*	3	12.0	11.0	29.0	*Campylobacter rectus*	1	100.0
*Rothia mucilaginosa*	3	6.0	4.5	8.0	*Capnocytophaga gingivalis*	1	1.0
***Fusobacterium nucleatum***	2	75.0	62.5	87.5	*Capnocytophaga ochracea*	1	30.0
***GPAnB*** ^b^	2	25.0	22.5	27.5	*Capnocytophaga spp*	1	30.0
***Veillonella parvula***	2	50.2	25.3	75.1	*Enterobacter amnigenus*	1	500.0
*Streptococcus anginosus*	2	55.0	32.5	77.5	*GNAeB* ^c^	1	30.0
*Streptococcus salivarius*	2	5.5	4.8	6.3	*Kocuria spp*	1	800.0
*Streptococcus sanguinis*	2	27.5	26.3	28.8	*Micrococcus flavus*	1	2.0
*Bacillus spp*	2	4.0	3.5	4.5	*Neisseria elongata*	1	20.0
*Capnocytophaga sputigena*	2	5.1	2.7	7.6	*Neisseria meningitidis*	1	0.1
*Gemella morbillorum*	2	110.0	65.0	155.0	*Neisseria perflava*	1	1.0
*Neisseria macacae*	2	1.2	0.7	1.6	*Pectobacterium spp*	1	400.0
***Actinomyces spp***	1	10.0			*Candida albicans*	1	1.0
***Eubacterium yurii***	1	20.0					

Microbial counts of each strains detected from different samples are presented in their median, 25% and 75% quartiles (×10^3^ CFU/ml). The boldfaced strains are anaerobic. ^a^*GPAeB*: Gram positive aerobic bacilli.

^b^*GPAnB*: Gram positive anaerobic bacilli.

^c^*GNAeB*: Gram negative aerobic bacilli.

25%: the first quartile.

75%: the third quartile.

The microbial loads of each species varied among different samples. *Streptococcus intermedius* and *Gemella morbillorum* had the highest median counts, whereas *Slackia exigua*, *Kocuria spp., Prevotella intermedia, Streptococcus intermedius* and *Enterobacter amnigenus* were abundant and showed higher counts in some samples (Table 1).

### Microbiological findings among different PPDs

**Figure 2:**
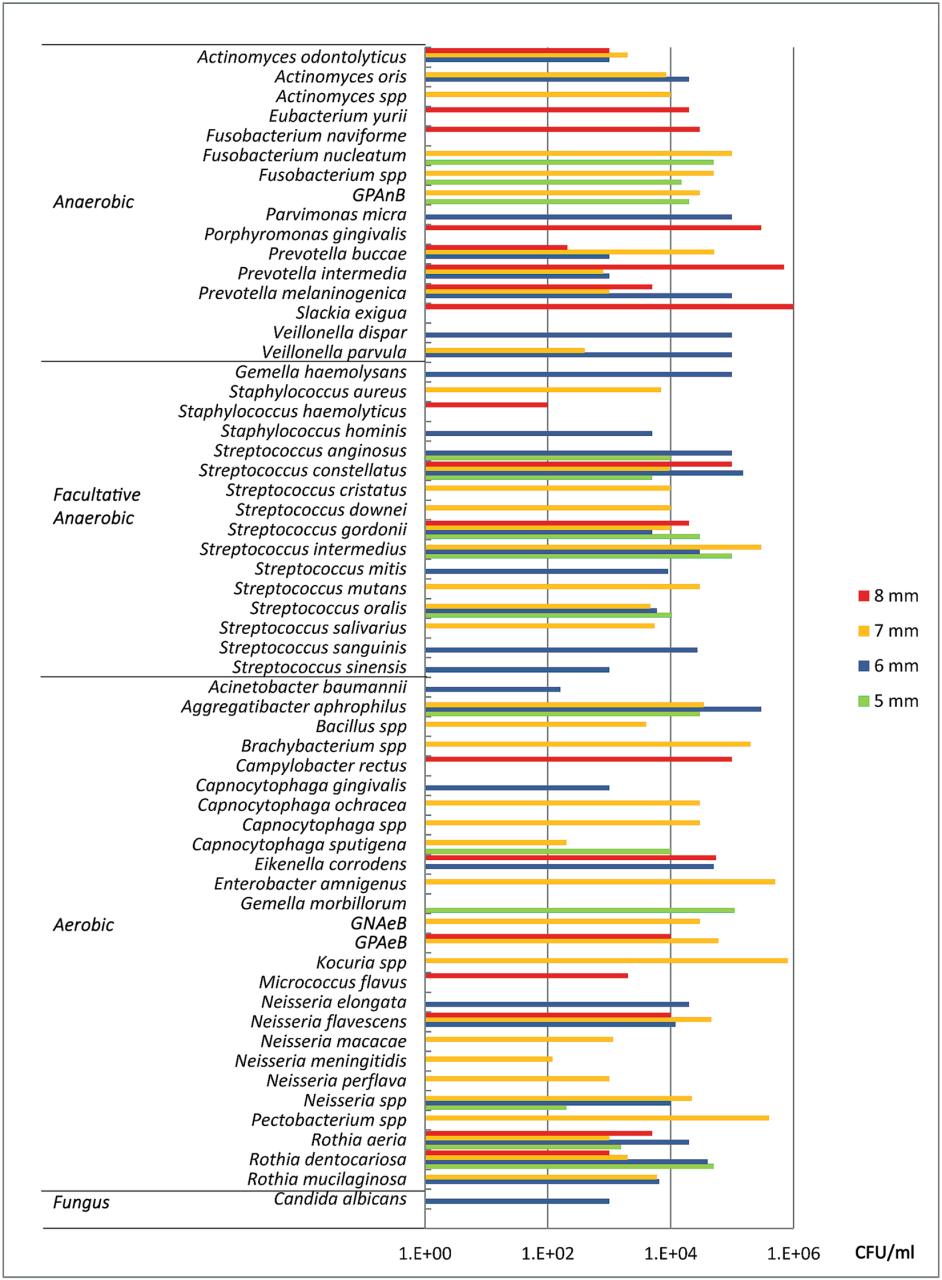
Median microbial counts of each strains found in different peri-implant pockets, detected by MALDI-TOF MS. Higher counts of anaerobic bacteria, such as *S. exigua, P. intermedia, and P. gingivalis*, were present in deeper pockets (8 mm), whereas some aerobics, such as *Kocuria spp., Enterobacter amnigenus and Pectobacterium spp*., were found to be high in 7-mm PPDs. Otherwise, the distribution of facultative anaerobic and aerobic microorganisms was dispersed.

The deepest sites of the tested peri-implantitis pockets were measured from 5 to 8 mm. Microbial strains and their median counts were analyzed among different PPDs. Higher counts of anaerobic bacteria, such as *S. exigua, P. intermedia*, and *P. gingivalis*, were present in deeper pockets (8 mm), whereas some aerobics, such as *Kocuria spp*., *Enterobacter amnigenus* and *Pectobacterium spp*., were found to be high in 7-mm PPDs. Otherwise, the distribution of facultative anaerobic and aerobic microorganisms was dispersed. (Fig. 2) The median counts of different microbial types among different PPDs are presented in Table 2. There were no statistically significant differences of anaerobic, facultative anaerobic and aerobic counts between each pair of PPD groups (*p* > 0.05). The highest median percentage of anaerobic microbiota was found in 8-mm PPDs, whereas that of aerobic microbiota was in 5-mm pockets (Table 3). There were no statistically significant differences of anaerobic, facultative anaerobic and aerobic microbial percentages among groups, nor between each pair of PPD groups (*p* > 0.05).

**Table 2:** Microbial counts of different microbial types among different depths of peri-implant pockets are presented in median, 25% and 75% quartiles (CFU/10^4^).

Microbiota (CFU/10^4^)	Median (25%, 75%)				
Deepest PPD	5 mm	6 mm	7 mm	8 mm	All specimens
Anaerobic	5.0 (3.5, 6.5)	20.0 (11.5, 20.7)	5.2 (4.9, 10.0)	102.8 (51.7, 153.9)	6.6 (2.8, 17.0)
Facultative anaerobic	8.3 (4.5, 12.2)	10.7 (9.1, 26.9)	10.7 (7.2, 11.4)	11.0 (5.5, 16.5)	10.7 (5.6, 17.5)
Aerobic	15.7 (8.8, 22.5)	4.0 (3.7, 21.6)	8.4 (4.6, 10.2)	12.2 (11.7, 12.6)	9.3 (3.9, 17.2)
Fungus	ND	0.1 (0.1, 0.1)	ND	ND	0.1 (0.1, 0.1)
Total	29.0 (16.8, 41.1)	35.4 (25.0, 68.8)	22.4 (22.3, 24.3)	126.0 (80.8, 171.1)	29.9 (20.4, 65.5)

There are no statistically significant differences of anaerobic, facultative anaerobic and aerobic microbial counts among groups, nor between each pair of PPD groups (*p* > 0.05). ND: not detected.

**Table 3:** Microbial percentages of different microbial types among different depths of peri-implant pockets are presented in median, 25% and 75% quartiles.

Microbiota (%)	Median (25%, 75% quartiles)				
Deepest PPD	5 mm	6 mm	7 mm	8 mm	All specimens
Anaerobic	29.1% (22.1%, 36.2%)	20.5% (20.0%, 40.3%)	21.4% (5.2%, 22.0)	48.3% (25.0%, 71.5%)	21.0% (12.6%, 43.6%)
Facultative anaerobic	21.5% (17.2%, 25.8%)	42.1% (36.2%, 46.7%)	32.3% (16.2%, 44.0)	30.8% (15.4%, 46.2%)	31.3% (15.4%, 45.7%)
Aerobic	49.4% (46.6%, 52.2%)	27.5% (18.5, 32.9%)	45.7% (34.6%, 78.6)	20.9% (13.1%, 28.8%)	37.5% (23.0%, 48.0%)
Fungus	ND	0.7% (0.7%, 0.7%)	ND	ND	0.7% (0.7%, 0.7%)

There are no statistically significant differences of anaerobic, facultative anaerobic and aerobic microbial percentages among groups, nor between each pair of PPD groups (*p* > 0.05). ND: not detected.

## Discussion

Using the MALDI-TOF MS technique, we were able to detect the bacterial diversity of at least 59 different living strains from 12 samples, and to calculate the bacterial loads and the compositions at the same time. MALDI-TOF-MS has emerged as a potential tool for microbial identification and diagnosis and has barely been used for bacterial identification in peri-implantitis. It makes a major difference in this study because this method is rapid, sensitive, and economical in terms of both the labor and cost involved.

MALDI-TOF MS-based identification has also introduced a new era in anaerobic microbiology. It is well known that anaerobic bacteria often predominate in peri-implantitis or periodontitis. The isolation and identification of anaerobic bacteria by conventional methods is often cumbersome and time-consuming. Molecular methods have been developed in the laboratory and are characterized by high sensitivity and specificity, but they are often expensive^21–23^. Using MALDI-TOF MS, Stîngu *et al*. tested 75 strains isolated from subgingival biofilms of 33 patients with aggressive periodontitis and 9 reference strains. The identifications were compared with sequence analysis of the 16S ribosomal RNA (rRNA) gene in detail. They found that almost all the spectra of the same species clustered together with very few exceptions. The capability of MALDI-TOF MS to distinguish between *Prevotella intermedia* and *Prevotella nigrescens*, two phenotypically indistinguishable species, was found^13^. The capability to identify the intra-species diversity of *Fusobacterium spp*., *Clostridium spp*., *Bacteroides spp*. and other Gram-positive anaerobic cocci were found in other studies^24^. Furthermore, a combination of MALDI-TOF MS with powerful classification algorithms provided a useful tool to differentiate and identify 14 oral *Actinomyces* species, which is often difficult and time-consuming by conventional methods^25^. Within the limitation, MALDI-TOF MS is capable to distinguish cultivable anaerobic pathogen to an exact state.

The current database of MALDI-TOF MS bacterial detection comprises over 1,000 species, and the database is continuously growing. One of the advantages of using MALDI-TOF MS is to explore new microbial strains that were unrevealed or unfamiliar with peri-implantitis or other oral infections. For example, we were able to identify the quantity of *Slackia exigua* from one deep and putative peri-implantitis pocket. *Slackia exigua* is a poorly growing pathogen in periodontitis and periapical infections and has proven to be difficult to culture and is unreactive in conventional biochemical tests, but it was exclusively reported by the analysis of 16S rRNA gene sequencing^26^. Coincidently, Tamura *et al*. analyzed bacterial flora associated with peri-implantitis using the obligate anaerobic culture technique and 16S rDNA sequencing and demonstrated that the sulcus around oral implants with peri-implantitis harbors high levels of asaccharolytic anaerobic gram-positive rods (AAGPRs), such as *Slackia exigua* and *Eubacterium spp*., and gram-positive anaerobic rods, suggesting that AAGPRs may also play an important role in peri-implantitis^27^.

Indeed, 16S rRNA gene sequencing is ubiquitous in molecular techniques and allows for the detection of previously uncultivated and unknown bacteria. A recent study has shown that overall, between 16S gene-based and clinical identities, the genus-level concordance rate is 96% and the species-level concordance rate is 87.5%^28^. Results from this study demonstrated a genus-level identification rate of 95.2% and a species-level rate of 82.5% by MALDI-TOF MS, which is similar to the 16S gene-based method. In the present study, we found 47 species, 8 genera and 1 *Candida*, representing 5 phyla and 1 fungus. This finding coincides with another *in situ* investigation by de Melo *et al*., in which oral bacteria on titanium implant surfaces were identified by 16S rDNA sequencing. A total of 29 genera were identified, representing exactly the same phyla that we found: *Firmicutes, Proteobacteria, Fusobacteria, Bacteroidetes, Actinobacteria* and *Candida*^29^.

In general, the results from this study agreed with previous findings and showed that some *Streptococcus spp*., *Actinomyces spp*. and *Fusobacterium spp*. are common to sites with peri-implantitis. However, the distribution of bacteria detected by MALDI-TOF MS differed greatly from implant to implant. The large variation in regard to the microbial profiles, similar to previous studies, makes interpretations of a correlation between disease progression and microbial profiles difficult^9,30–32^. To overcome the variation of bacterial profile related to disease and the uncertainty in anaerobic culturing proceeding MALDI-TOF MS analysis, a large scale of sampling seems necessary before conclusive remark is made. Other methodological details might also be considered, such as using different transportation medium or extending the incubation period^25^, although some studies found that both the culture conditions and the culture time did not affect microbial identification by MALDI-TOF MS^33,34^.

However, it is difficult to collect samples from peri-implant sulci because of structural problems. In this study, paper points were used by inserting them into peri-implantitis pockets, and the collection of crevicular fluid and microbiota was expected. The microbial composition in peri-implantitis pocket fluid samples taken by paper points might have differed from those of curette samples taken from implant surfaces or soft tissues of the pocket. Gerber *et al*. compared the differences and found significantly higher total DNA bacterial counts and higher proportions for 28 out of 40 species tested in fluid samples^35^. These species comprised some important periodontal pathogens, such as *Treponema forsythia*, *Treponema denticola*, and *Aggregatibacter actinomycetemcomitans*. Their results suggest that it could be difficult to collect samples from implants with curettes and that some bacteria are more prevalent in crevicular fluid and are not attached to implant surfaces. However, the adhesion of bacteria depends on surface characteristics, which is often involved in the initial bacterial colonization^36,37^. The microflora in the biofilm may be different in the crevicular fluid versus at the implant surface. Some bacteria, such as *P. gingivalis*, *P. intermedia* and *C. gracilis* in Gerber’s study, might have a preference for the implant surface or the soft tissue, so they exhibit less prevalence in fluid samples. Therefore, these bacteria might have been underestimated in this study.

In addition, the quality and reliability of the bacterial identification depends on the quality and quantity of reference spectra present in the database. The limitation of the technology is that the identification of new isolates is possible only if the spectral database contains peptide mass fingerprints of the type of specific strain. To date, some bacterial species known as pathogens to peri-implantitis and periodontitis, such as *Treponema forsythus, Treponema denticola* and *Aggregatibacter actinomycetemcomitans*, have not yet been included in database; therefore, their relationship to peri-implantitis could not be defined by MALDI-TOF MS. Database refinement and enrichment are essential elements of MALDI-TOF MS and will allow the method to increase its power as it is used more frequently and accurately^24,25,38^. In addition, in-house MALDI-TOF MS libraries using the spectra from reference and clinical isolates can be created to supplement the data base in the subsequent studies^39^.

Nowadays peri-implantitis is well defined as concomitant BOP and detectable bone loss^2,3,5^. Similar to periodontitis, severity of peri-implantitis varies. It is considered as moderate/severe peri-implantitis when implant sites present >2 mm bone loss, whereas on the other hand, mild peri-implantitis^2^. A study suggested that a higher extent of bone loss was often detected at the buccal compared with the other sites^40^. Radiographic detection of bone loss might not fully respond severity of peri-implantitis. With the limit of the study, disease severity was not taken into consideration.

The results from this study showed that *Streptococcus spp., Actinomyces spp. and Fusobacterium spp*. are common to sites with peri-implantitis. However, the distribution of bacteria differed greatly from implant to implant. With respect to diversified species in peri-implantitis, MALDI-TOF MS could be a potential method in developing oral microbiology in a more specific, easy, rapid and economical way. This preliminary study provides data for future study designs involving MALDI-TOF MS and peri-implantitis. The relation between bacterial profiles and disease progression might be gradually clarified as the MALDI-TOF MS database is increased and comprehensive data is approached.

## Data Availability

All data generated or analysed during this study are included in this published article.
